# Formulation and Evaluation of Silymarin-Loaded Chitosan-Montmorilloite Microbeads for the Potential Treatment of Gastric Ulcers

**DOI:** 10.3390/jfb9030052

**Published:** 2018-09-10

**Authors:** Ameya Sharma, Vivek Puri, Vandita Kakkar, Inderbir Singh

**Affiliations:** 1 Department of Pharmaceutics, Chitkara College of Pharmacy, Chitkara University, Patiala 140401, India; ameya.nancy91@gmail.com (A.S.); vivekpuri92@gmail.com (V.P.); 2 University Institute of Pharmaceutical Sciences, Panjab University, Chandigarh 160101, India; vanditakakkar@yahoo.co.in

**Keywords:** Microbeads, silymarin, chitosan, montmorillonite, gastric ulcers

## Abstract

Silymarin-loaded mucoadhesive microbeads of Chitosan-MMT were developed using the ionotropic gelation technique. Characterization of the microbeads was performed by DSC, XRD, SEM, and FTIR techniques. In vitro mucoadhesion and drug release studies; gastroprotective studies including the measurement of ulcerative index; the determination of gastric wall mucus; and the determination of percentage protection, biochemical, and histopathological studies were also performed. Microbeads batches were evaluated for particle size (120–140 µm), actual drug content, (49.36–58.18%) and entrapment efficiency (72.52–92.39%).Biochemical estimation of myeloperoxidase was found to be 0.10–0.75 µmoles/g/tissue. Significant reduction in the ulcerative index showed the gastroprotective effect of the formulation. Silymarin-loaded beads of Chitosan-MMT were found to exhibit good mucoadhesion and efficient release of the drug, and were found to be a promising drug carrier system for the treatment of gastric ulcers.

## 1. Introduction

Innumerable oral delivery systems acting as controlled release systems for the delivery of pharmaceutical actives have been developed. Gastroretentive drug delivery systems could be developed by employing raft, bio adhesion, floating, swelling, low/high density, and expanding technologies. Improved bioavailability, controlled release, and enhanced therapeutic activity can be further achieved by developing the gastroretentive drug delivery systems. Mucoadhesion is a common phenomenon for developing gastroretentive drug delivery systems. Concentration, molecular weight, type, and number of hydrolyzing groups in the mucoadhesive polymer and the presence of additives are important for developing mucoadhesive drug delivery systems. Various natural and synthetic polymers are used for developing mucoadhesive formulations [1]. Chitosan is a nontoxic, biocompatible, and biodegradable linear polysaccharide consisting of *β* (1–4)-linked 2-amino-2-deoxy-d-glucose (d-glucosamine) and 2-acetamido-2-deoxy-d-glucose (N-acetyl-d-glucosamine) units. It is the N-deacetylated derivative of chitin. Different grades of chitosan are available depending on the amount/extent of deacetylation. Interesting properties of chitosan include wound healing, antimicrobial mucoadhesivity, biocompatibility, and low toxicity [2].

Montmorillonite (MMT) is smectite group clay and is composed of silica tetrahedral sheets layered between alumina octahedral sheets. It has mainly industrial applications, which are due to its large specific surface area, good adsorption ability, cation exchange capacity, adhesive ability, and drug carrier capability [3]. Chitosan clay composites have been used for developing tablets [4], films [5], and hydrogels [6], and for microparticles [7] for drug delivery systems.

*Silybum marianum*, which is commonly familiarized as milk thistle, belongs to the family Asteraceae; it comes under one of the primeval experimented plants in ancient times and was used to test liver and gallbladder disorders, including the treatment of jaundice, hepatitis, and cirrhosis; it was also used for the toxin poisoning and for the protection against *Amanita phalloides* mushroom. Silymarin is the active constituent of the plant, which is a standardized extract and has Silymarin flavonoligans of around 70% to 80% (silydianin, silychristin, isosilybin A & B, silybin A & B) and flavonoids (quercetin and taxifolin); the remaining part (20–30%) consists of a chemically undefined fraction that is comprised of polyphenolic compounds (polymeric and oxidised) [8].

The objective of this work was to formulate and evaluate Silymarin-loaded Chitosan-MMT microbeads. The formulated microbeads were evaluated for size, entrapment efficiency, and in vitro mucoadhesion. FTIR, SEM, DSC, and XRD analyses were performed to characterize the microbeads. In vitro drug release data was fitted to various drug-release kinetic models to understand the mechanism of the drug release from the microbeads. Suitable animal studies were performed to evaluate the gastroprotective property of the formulation.

## 2. Results and Discussion

### 2.1. Analytical Method

The standard plot of Silymarin was prepared in 0.1 N HCl at λmax 290 nm.

The equation for linearity is Y = 0.064x + 0.001; R^2^ = 0.997 for 0.1 N HCl.

Limit of detection (LOD) was found to be 1.886 µg/mL. Limit of quantification (LOQ) was found to be 5.842 µg/mL.

### 2.2. Formulation and Evaluation of Silymarin Loaded Chitosan Microbeads

Preliminary batches of chitosan microbeads were prepared for optimizing the concentration of chitosan (Sigma Aldrich, St Louis, MO, USA) and tripolyphosphate (crosslinking agent) (Loba Chemie, Mumbai, India). Chitosan (4% w/v) and tripolyphosphate (10% w/v) in formulation batch F6 were found to be optimum, as microbeads formed were uniformly round with no tailing. Moreover, the F6 batch exhibited maximum percent yield and entrapment efficiency as mentioned in Table 1.

Based upon the ratios of chitosan and MMT, the Silymarin-loaded chitosan-MMT mucoadhesive bead formulations were coded as CMMTB10, CMMTB11, CMMTB21, CMMTB31, CMMTB41, and CMMTB51 respectively.

### 2.3. Characterization Chitosan-MMT Beads of Silymarin

#### 2.3.1. Bead Average Diameter

**Figure 1:**
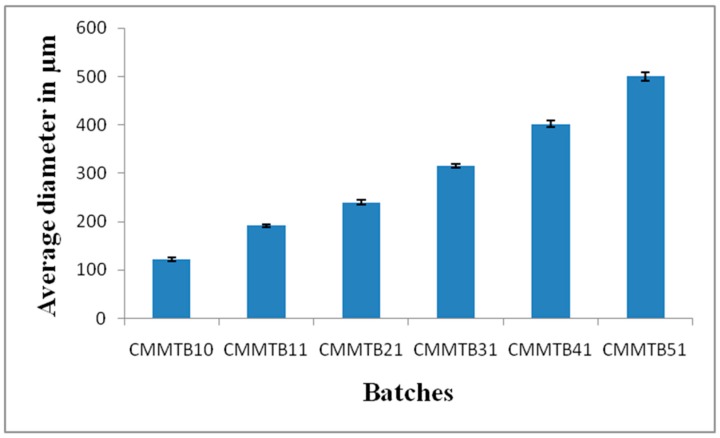
Average diameter of Silymarin-loaded Chitosan-MMT microbeads of different batches.

The mean diameter of Chitosan-MMT beads is shown in Figure 1. The mean diameter of the microbeads was found to range between 120 and 500 µm. As the concentration of the polymer increased, the size of the microbeads was found to increase proportionately. This can be attributed to increase in viscosity of the polymer solution, which increased the polymer concentration. Behin et al. reported that with increase in polymer concentration in beads, the particle size of beads also increases [9].

#### 2.3.2. Determination of Entrapment Efficiency (EE)

Entrapment efficiency was found to be associated with the size of the beads, which was further dependent upon the concentration of the polymer used. Incorporation of MMT enhanced the drug entrapment because of the electrostatic interaction between Chitosan and MMT as mentioned in Table 2. Takka et al. formulated bovine serum albumin-loaded beads prepared using the ionotropic gelation technique and reported that the chitosan concentration significantly influenced the particle size, actual drug content and the entrapment efficiency of the beads [10].

#### 2.3.3. FTIR Analysis

Pure Chitosan spectra showed peak at 1031.25 cm^−1^ assigned to the presence of saccharide structure. Peaks at 3450.25 cm^−1^, 1582.12 cm^−1^ and 1375.34 cm^−1^ were assigned to stretching vibrations of OH, amide I, and amide III respectively. FTIR spectra of MMT exhibited peaks at 3428.35 cm^−1^ due to –OH stretching and at 1596.75 cm^−1^ due to NH primary and secondary amines and bending vibration of hydroxyl group. Band at about 1034.26 cm^−1^ corresponds to C–O stretching.

**Figure 2:**
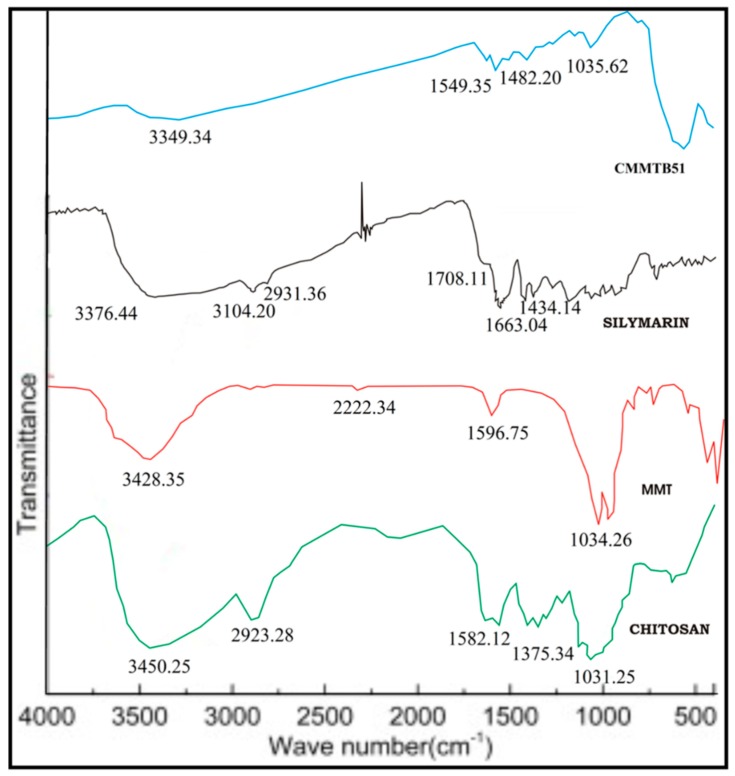
Layout representing the FTIR spectra of CMMTB51, Silymarin, MMT, and Chitosan.

The FTIR spectra of Silymarin showed peaks at 3376.44 cm^−1^ corresponding to phenolic (OH) vibrations, 1663.04 cm^−1^ corresponds to mixed (C=O) amide and (C=C) vibrations, and 1434.14 cm^−1^ is attributed to the symmetric aromatic ring stretching vibration (C=C ring). Chitosan-MMT beads-loaded Silymarin showed peaks at 1549.35 cm^−1^ and 1482.20 cm^−1^ indicating C=C aromatic, N–H/C–H stretching, and CH_2_ wagging coupled with OH groups of Chitosan. The absence of characteristic peak of drug indicates the successful entrapment of drug within the polymeric matrix of microbeads as shown in Figure 2.

#### 2.3.4. DSC

**Figure 3:**
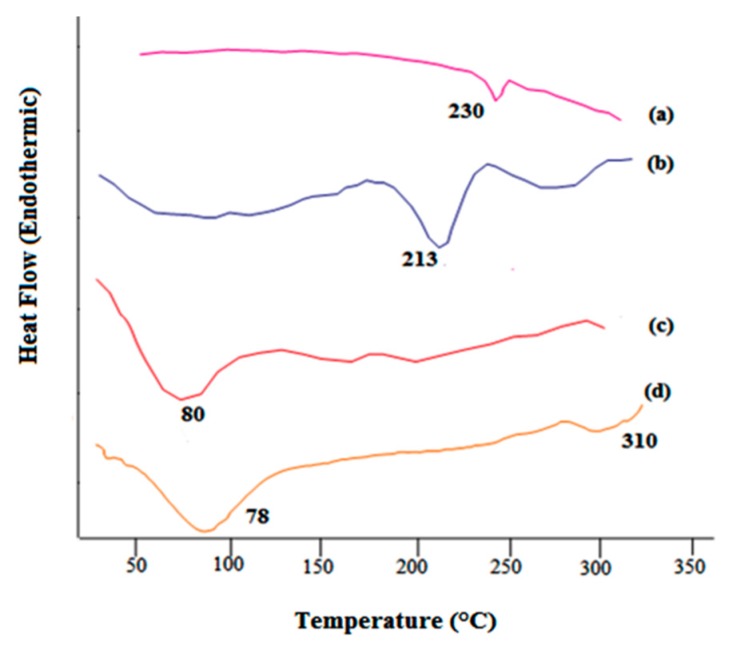
DSC thermogram of (**a**) drug, (**b**) CMMTB51, (**c**) MMT, and (**d**) Chitosan.

DSC is a useful technique for determining the changes in thermal transitions. MMT showed broad endothermic peak at 80 °C. It has been investigated that complexation alters the crystallanity of two polymers leading to formation of broad peak. DSC of the drug showed a sharp peak at 230 °C. As investigated in Figure 3, Chitosan showed first endothermic peak at 78 °C and second endothermic peak at 310 °C. Abdul reported the appearance of exothermic peaks of pectin and chitosan in the physical mixture at 240 and 308 °C respectively. However, in interpolymer complex mixture of the two polymer, the shifting of the peaks to lower temperatures (213 °C and 260 °C) indicates the interactions between the two polymers and is considered as a proof of their complexation [11].

#### 2.3.5. Scanning Electron Microscopy (SEM)

**Figure 4:**
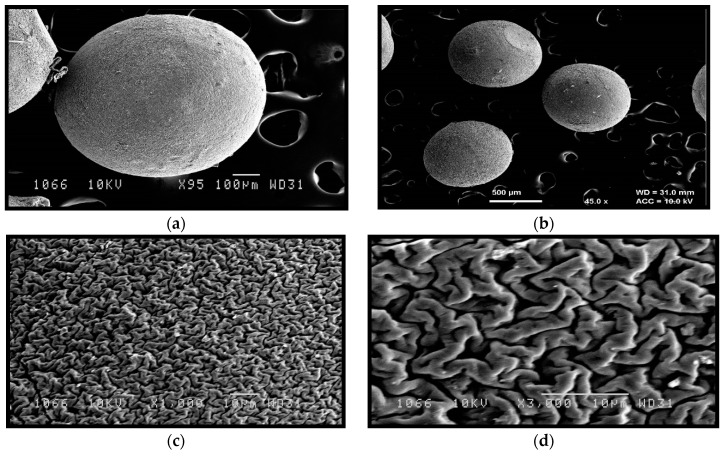
SEM images of formulated beads at different magnification value (**a**) at 95×, (**b**) 45×, (**c**) 1000×, and (**d**) 3000×.

SEM analysis was performed to study the morphological and surface characteristics of the microbeads. The microbeads prepared were established to be sphere-shaped (Figure 4). Surface smoothness was observed with Silymarin-loaded Chitosan-MMT mucoadhesive microbeads. Striation on the surface can be related to the release of the drug from within the microbeads.

#### 2.3.6. X-ray Diffraction Studies

**Figure 5:**
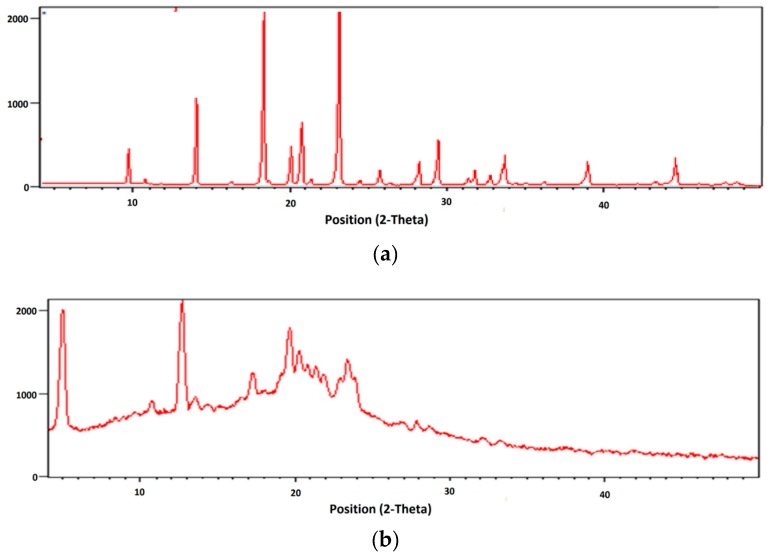
X-ray Diffraction pattern of (**a**) Silymarin- and (**b**) Silymarin-loaded Chitosan-MMT beads.

The X-ray diffraction pattern of Silymarin depicts its crystalline nature. However, absence of characteristic peaks of the drug in the XRD diffractogram of microbeads indicates entrapment of drug within the polymeric matrix of microbeads (Figure 5).

### 2.4. In Vitro Mucoadhesive Property

Formulated microbeads of chitosan-MMT established a good mucoadhesive property (Table 3). It was noted that the mucoadhesion of the polymer increased with the increase in the concentration of the polymer. The mucoadhesive property of the formulation direct correlated with the concentration of the mucoadhesive polymer. Patel J. et al. reported that formulation batch containing high concentration of polymer adhered more strongly to gastric mucus layer and could also remain in the gastro intestinal tract for an extended period of time [12].

### 2.5. In Vitro Drug Release

**Figure 6:**
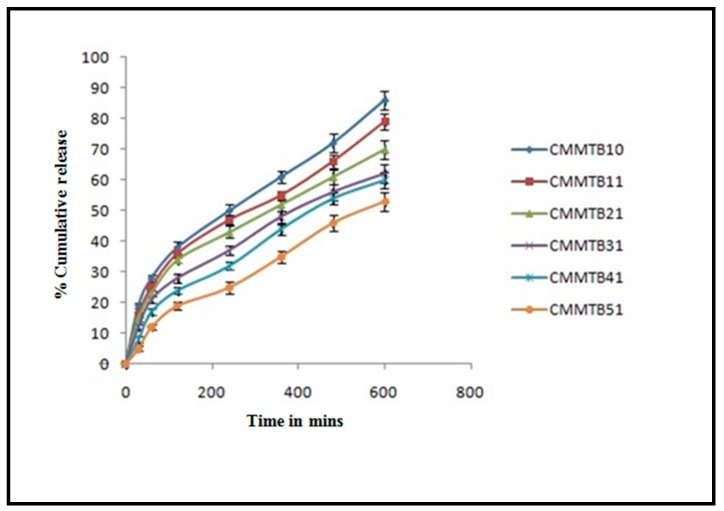
In vitro release profile of Silymarin from different batches of microbeads.

In vitro drug release study for CMMTB10, CMMTB11, CMMTB21, CMMTB31, CMMTB41, and CMMTB51 was carried out for 10 h. CMMTB51 showed highest percent cumulative release up to 10 h. CMMTB10 exhibited 86% of the drug release in 10 h, and CMMTB11 showed 79% of the drug release in 10 h, as it contains Chitosan and MMT in ratio of 1:1. As the concentration of Chitosan is increased, the percent cumulative release was found to be decreased. CMMTB51 exhibited 58% of the drug release in 10 h. The CMMTB51 showed controlled drug release. Results from the release study depict that the concentration of Chitosan in beads is a critical factor behind the release of drug as shown Figure 6. Chitosan produced a partially hydrophobic matrix, which has less affinity for water resulting in retardation of drug release form the formulation. Abdelaal et al. also conducted study on chitosan hydrogels using 5-fluorouracil as a model drug and concluded that release of drug becomes slower when the concentration of polymer increases [13].

The in vitro drug release (kinetic modeling) data of the formulated batches is shown in the Table 4. The value of n (release exponent) 0.45 ˂ n ˂ 0.89 indicates Non-Fickian drug release transport. The value of n was found to be in the range of 0.47 to 0.730 for all the formulations (CMMTB10-CMMTB51) except those that follows first order kinetics (r^2^ = 0.98). This indicated that the drug release from the formulation follows diffusion and the erosion of polymer (anomalous Non-Fickian drug release behavior). When the formulation is in contact with the dissolution media, the media penetrates the lattice network of the polymer leading to polymer chain disentanglement causing polymer erosion and subsequent release of the drug molecules. According to an another theory explaining the drug release mechanism, the glass-rubbery change of the polymeric dosage form in the media causes an increase in polymeric chain mobility such that the network mesh of the polymer enlarges/erodes, thus permitting the molecules of the drug to pass through and dissolve completely in the gel layer.

### 2.6. Gastroprotective Studies

Ethanol causes acute hemorrhagic gastric erosion, leading to mucosal edema and cellular exfoliation symbolizing acute inflammatory reaction; ethanol can easily penetrate the gastric mucosa. The model group depicted various macroscopic morphological variations. A pathology score was studied to evaluate whether the pretreated beads loaded with Silymarin could guard against mucosal injury caused due to ethanol. As shown in the Table 5, the model group displayed has a significant increase of ulcer index (85 ± 3.12) in comparison to control group (*p* < 0.05); the group already treated with omeprazol or Silymarin beads can decrease the ulcer index (*p* < 0.05) and increase the protection percentage significantly. Ethanol, when administered orally, decreases the production of mucus considerably compared to normal group (*p* < 0.05), but if pretreated with omeprazol or beads loaded with Silymarin, elevates the mucus in the gastric mucosa. Qin et al., reported that microspheres that are mucoadhesive and have alkaloids can decrease gastric injuries by shortening the ulcerative index, increasing the protection percentage, and elevating the mucus amount in mucosa in an animal model of ethanol-induced gastric mucosal injury [14].

#### Myeloperoxidase (MPO)

Myeloperoxidase is seen in neutrophil granulocytes, which produces hypohalous acids to produce antimicrobial activity. Myeloperoxidase has heme pigment, which is responsible for its green color in neutrophils. The level of myeloperoxidase was significantly increased in disease control group 2.675 ± 0.684 compared to the beads group. This causes the peroxidation of lipids, which increases the myeloperoxidase activity. The level of total Myeloperoxidase in native control group (0.105) was significantly decreased compared to the beads of high and low dose as shown in Table 5. The variations in gastric accretion of leukocytes followed by the ethanol produced lesions were characterized by calculating gastric myeloperoxidase activity, which was considerably elevated in comparison to the positive control. Tariq et al. reported the protective effects of simvastatin against chemically induced gastric ulcers and reduced oxidative stress. The level of myeloperoxidase in native group decreased compared to the formulation dose [15].

## 3. Materials and Methods

### 3.1. Materials

Materials consisted of Silymarin, chitosan (medium molecular weight, 85% deacylated), and Montmorillonite (MMT) (Sigma-Aldrich, St. Louis, MO, USA), Tripolyphosphate (TPP) (Loba Chemie, Mumbai, India). All other chemicals and reagents used in study were of analytical grade.

### 3.2. Methods

#### 3.2.1. Analytical Methods

##### Standard Plot in 0.1 N HCl

Stock solution of Silymarin was prepared by dissolving 10 mg of the drug in 10 mL of methanol: 0.1 N HCl (1000 µg/mL; n = 6). The stock solution was diluted serially to obtain a concentration range of 2 µg/mL–10 µg/mL, which was analyzed spectrophotometrically at λ_max_ of 290 nm using 0.1 N HCl as blank. The observed absorbance values for the respective dilutions were plotted against corresponding concentrations, and straight lines passing through origin were obtained.

Linearity: 50 µg/mL solution of Silymarin was prepared (n = 6) in above-mentioned solvent systems. From these stock solutions, the different concentrations (2 µg/mL–10 µg/mL) were prepared and analyzed in chloroform: methanol and 0.1 N HCl.

##### Limit of Detection (LOD)

Limit of detection is defined as the lowest amount of analyte in a sample that can be detected but not necessarily quantitated as an exact value. Six identical samples were analyzed for the determination of LOD. LOD was determined by LOD = 3.3 σ/S in which *σ* was obtained from standard deviation of intercepts of the regressed lines and was the average slope of the regressed line.

##### Limit of Quantification (LOQ)

Limit of quantification is defined as the lowest concentration at which the precision expressed by relative standard deviation is better than 20% and inaccuracy expressed by relative difference of the measured and true value is lower than 20%. Six identical samples were analyzed for the determination of LOQ. LOQ was determined basically for impurity analysis, LOQ based on the standard plot, according to which LOQ = 10 σ/S

Where, *σ* was obtained from standard deviation of intercepts of the regressed lines and *S* was the average slope of the regressed line.

#### 3.2.2. Preparation of Chitosan-MMT Beads of Silymarin

Ionotropic gelation method was used for the formulation of chitosan-MMT beads. Chitosan and MMT in ratio of (1:0, 1:1, 2:1, 3:1, 4:1, 5:1) were used for preparing beads. An aqueous solution of Chitosan was prepared by dissolving chitosan in glacial acetic acid (5% w/v) with continuous stirring using a magnetic stirrer until the chitosan was completely dissolved. Silymarin (50 mg) was then added to the chitosan solution. MMT was dispersed in distilled water and stirred for 20 min. The chitosan solution (containing Silymarin) was then added to the MMT dispersion, and the mixture was stirred at 2000 rpm for 1 h. The Chitosan/Silymarin/MMT solution was added drop wise into TPP (10% w/v) containing glutaraldehyde (5% w/v) using a 22 gauge syringe employing constant pressure. The Silymarin-loaded chitosan-MMT beads were washed thoroughly with distilled water and dried in an oven at temperature not exceeding 40 °C. The dried beads were stored in desiccator for further use. The composition of Silymarin loaded Chitosan- MMT beads is shown in Table 6.

#### 3.2.3. Characterization of Chitosan-MMT Beads

##### Bead Average Diameter

Average diameter of beads was calculated by calibrated eye piece micrometer of optical microscopy. The smear formed by the dispersion of microbeads in liquid paraffin was viewed under compound microscope. A calibrated eye micrometer was used to calculate the size of the size of 10 beads for each batch of microspheres.

##### Determination of Entrapment Efficiency

For the determination of beads (10 mg), they were crushed in a mortar and pestle, which was made of glass, to prepare Chloroform: Methanol (10 mL) suspension. This was kept for stabilization overnight. The crushed beads were centrifuged at 1000 rpm, and Silymarin content was determined by dilution of supernatant using UV-Visible spectrophotometer at a wavelength of 290 nm. Entrapment efficiency (EE) was computed in accordance to the Equation (1):

(1) $$\mathrm{EE}\;\left(\%\right)=\frac{\mathrm{Mact}}{\mathrm{Mthe}}\times 100\%$$

in which M_act_ is the actual drug content in the beads and M_the_ is the theoretical amount of the drug in the beads calculated from the quantity added in the process. The data reported is mean ± SD of three determinations.

##### FTIR

Drug polymer interactions were checked by FTIR spectroscopy. FTIR spectra of the batch CMMTB51, Silymarin, MMT, and Chitosan were carried out using FTIR spectrophotometer (IFS 66/S, Alpha Bruker, Germany) in the reflectance mode with the wave number region 4000–650 cm^−1^.

##### Differential Scanning Calorimetry (DSC)

Differential scanning calorimetry (DSC) has been one of the widely used calorimetric methods with which the drug compatibility and compatibility of the ingredients have been studied. Samples of chitosan, MMT, Silymarin, and CMMTB51 were kept in a flat round bottomed aluminium pans and heated at temperature of 50 °C to 300 °C at a range of 10°/min with nitrogen purging (50 mL/min), in which alumina was used as the reference standard in a differential scanning calorimeter.

##### Scanning Electron Microscopy

The surface and shape characteristic of selected Silymarin loaded chitosan-MMT beads were investigated using scanning electron microscope (S 4300 SE/N, Hitachi, Tokyo, Japan). The samples were formulated by sprinkling the formulation lightly on a double adhesive tape, which was pasted on an aluminium stub. Then, the stub was coated with gold to achieve a thickness of just about 300 A° in a high vacuum evaporator to develop an atmosphere of argon with the help of gold sputter. The photomicrographics were taken with the help of SEM of the randomly selected coated samples.

##### X-Ray Diffraction

XRD was used to study the crystallization of polymers and drug. It is a highly potential and developed technique fundamentally having scattered intensity of an X-Ray beam, which is hitting the sample as a function of incident and scattered angle, polarization and wavelength or energy. The flexibility and non-destructive operational protocols of the technique exhibit chemical composition, but this technique also shows the crystallographic structure of the raw samples and the refined goods.

#### 3.2.4. In Vitro Measurement of Mucoadhesion Property

The non-digested food from the lumen of rat stomach was washed with deionized water furtherand placed in natural at 4 °C and utilized for 6 h. A provision was made during the tissue washing to avoid damage to the mucosal membrane.

The in vitro estimation of the mucoadhesive properties of Chitosan-MMT beads was carried out using rat stomach tissue. The rat stomach tissue was washed with physiological saline, and the tissue was attached on a microscopic slide. The pressure of 25 g was applied on the microscopic slide for 10 min to bring in contact thirty pre-swollen beads to the intestine. United State Pharmacopoeia (USP) disintegration apparatus (EI Products, Panchkula, India) was used to determine the mucoadhesiveness of the microcapsules by linking the formed slide with the gut to the apparatus. The particles were washed off under the reciprocating motion of disintegration apparatus in 900 mL test medium (0.1 N HCl, pH 1.2). The remaining particles attached were counted after 0.5, 1, 2, 4, and 6 h.

#### 3.2.5. In-Vitro Release

The in-vitro dissolution was performed for the Chitosan-MMT beads of Silymarin using USP II dissolution apparatus (Lab India, DS 8000, Mumbai, India) in 900 mL dissolution medium at 100 rpm, 37 ± 0.5 °C. The dissolution media with 0.1N HCl was used order to stimulate the change in pH along the GIT. The in vitro drug release experiments were performed in 0.1N HCl for 10 h. At regular time intervals, samples were taken out from media of dissolution and filtered using Whatman filter paper (0.22 µm). The absorbance was calculated using UV/visible spectrophometrically (AU-2701, Systronics, Mumbai, India) at 290 nm. The graph was plotted against collective percentage of the drug released vs. time.

The data of the dissolution obtained was integrated from a plethora of kinetic models, i.e., zero order; Higuchi; first order; Korsmeyer-Peppas; and Hixson-Crowell, which was used to establish the drug release mechanism. Kinetic studies involve comparing the regression (R^2^) values obtained to select the best fit model.

#### 3.2.6. Animal Study

The animal study protocol (1181/PO/Ebi/08/CPCSEA) was conducted according to the procedure approved by the animal ethics team of Chitkara College of Pharmacy, Chitkara University, Punjab. All the experimental studies were performed on the Wistar albino rats of either sex having a range of weight between 180 g and 220 g. The housing of all animals was done in the cages made of polypropylene in an air-conditioned environment of 25 ± 2 °C with light and dark cycle of 12:12-h, respectively. They were then given water and feed.

##### Induction of Gastric Ulcer

The SD rats were deprived of food but had free access to water for 24 h before ulcer induction. Gastric mucosal lesions were induced with absolute ethanol at a dose of 1mL by oral administration. Rats were divided into five groups randomly: native control, model control (ethanol), positive control-omeprazol (1 mg) + ethanol, beads (5 mg) + ethanol, and beads (20 mg) + ethanol. All the animals were treated with omeprazol or Silymarin beads orally once a day for 3 days and were fasted overnight with free access to water until experiment. The rats were sacrificed and their stomachs were removed [16].

##### Gastro Protective Studies

*Determination of Mucosal Lesion Index and Protection Percentage*

Percentage protection and mucosal lesion index (MLI) were determined by opening the greater curvature of rat stomach and rinsing it thoroughly with normal saline. MLI and percentage protection was calculated and numbered as follows: if hemorrhagic erosion is 1 mm; the mean mucosal lesion score was computed by adding the total MLI for each animal. Mean mucosal lesions score was the percentage of protection (%). This was computed in accordance to the Equation (2):

(2) Protection%=(MLImodel−MLItreated)×100% MLImodel

*Determination of Gastric Wall Mucus*

Along the greater curvature, stomach was opened and the gastric elements were firstly put together, centrifuged, and finally examined to determine the gastric juice pH of the supernatant using a 0.1 N NaOH sol and definite digital pH meter.

##### Biochemical Estimation

*Estimation of Myeloperoxidase (MPO) Activity*

Spectrophotometrical methods were used to determine the myeloperoxidase activity in the gastric mucosa. Homogenization of initially weighed tissue was done firstly (1:10 w/v) in 0.5% hexadecyltrimethyl ammonium bromide (ICN, Cleveland, OH, USA) in 50 mmol potassium phosphate buffer (pH 6.0) in the bath of ice before sonication for 20 s. Three cycles of freeze/thaw were carried out, and further sonication was done (20 s in an ice bath). The centrifugation of the samples was done at 17,000× *g* (5 min, 4 °C), and the assay of myeloperoxidase in the supernatant was done by integrating 0.1 mL of supernatant with 2.9 mL of 50 mmol/L potassium phosphate buffer (pH 6.0) having 0.167 mg/mL O-dianasidine dihydrochloride and 0.0005% hydrogen peroxide. UV-visible spectrophotometer (Systronics, Mumbai, India) was used to measure the change in absorbance at 460 nm for 4 min.

## 4. Conclusions

The objective of the work was to develop and examine mucoadhesive beads containing Silymarin for gastroretentive delivery. Silymarin, a metabolite of *Silybum marianum,* is an excellent antioxidant molecule, which we selected as the molecule of choice for gastroretentive delivery system. Moreover, Silymarin has been used and prescribed since ancient times and is mostly used as a treatment for gastro intestinal tract (GIT) problems and liver diseases. Silymarin-loaded beads were successfully prepared by ionotropic gelation technique. Silymarin beads containing Chitosan-MMT were used as adhesive polymers for the gastroretentive controlled release of the drug. The MMT was added to get stable, rigid, and discrete beads. The prepared beads were evaluated by FTIR, XRD, and DSC studies, which give an idea of interaction by showing characteristic peaks and difference in melting point. In vitro dissolution profiles showed the release was sustained for 10 h. Batch CMMTB10 containing Chitosan alone provides delayed release of the drug from the formulation, whereas batch CMMTB51 gives the controlled release. All the batches showed mucoadhesive properties, but the batch containing a high amount of chitosan CMMTB51 offers maximum percentage mucoadhesion. The beads were further evaluated by SEM to analyze their morphology. SEM analysis of batch CMMTB51 showed that the beads were discrete and spherical in shape and the drug entrapment was successful. The entrapment efficiency of the microbeads was found to range between 72.52% ± 2.53% and 92.39% ± 4.02%. Silymarin beads followed the Higuchi model of release, suggesting a linear correlation between percent drug release and the square root of time. Additionally, the biochemical evaluation was established for the antioxidant potential of Silymarin beads, resulting in a significant decrease in myeloperoxidase level. Similarly, results were evidenced from gastroprotective studies and histological studies, which showed positive effects. Formulation with the higher concentration of chitosan showed results within all evaluated parameters and is considered as an ideal formulation. This investigation verified that the formulation was capable of achieving gastroretentive delivery of Silymarin.

## Figures and Tables

**Table 1:** Characterization of Chitosan microbeads of Silymarin.

Batches	Beads Shape	% Yield	EE (%)
F1	Oval and irregular	30.75	46.71 ± 2.24
F2	Oval and irregular	42.89	52.20 ± 1.91
F3	Oval with tailing	50.33	55.68 ± 1.38
F4	Round with tailing	62.69	60.54 ± 2.25
F5	Round with less tailing	68.23	73.97 ±1.19
F6	Round and uniform	78.86	81.34 ± 1.29
F7	Oval with less tailing	52.43	54.71 ± 2.74
F8	Oval with less tailing	56.81	55.84 ± 0.82
F9	Oval with less tailing	58.76	57.52 ± 1.54

**Table 2:** Entrapment efficiency of different formulation batches of Chitosan-MMT microbeads of Silymarin.

BATCHES	E.E (%)
CMMTB10	72.52 ± 2.53
CMMTB11	78.81 ± 1.09
CMMTB21	83.55 ± 2.99
CMMTB31	86.49 ± 3.62
CMMTB41	89.20 ± 2.96
CMMTB51	92.39 ± 4.02

**Table 3:** Percentage mucoadhesion of various chitosan-MMT beads of Silymarin.

% Mucoadhesion After (h)	CMMTB10	CMMTB11	CMMTB21	CMMTB31	CMMTB41	CMMTB51
0.5	20.19	26.23	30.26	32.18	42.34	55.12
1	13.56	19.17	25.34	28.32	35.14	48.65
2	11.72	15.28	21.67	24.87	30.32	43.21
4	8.10	13.85	17.15	20.54	24.45	37.78
6	6.67	10.40	14.423	16.25	20.76	31.43

**Table 4:** Release kinetic study of different formulations.

Batches	Zero Order		First Order		Higuchi Model		Hixon Crowell Model		Korsmeyer Peppas Model		
	r^2^	k_0_	r^2^	k_1_	r^2^	k_H_	r^2^	k_HC_	r^2^	n	k_KP_
CMMTB10	0.984	0.109	0.984	−0.109	0.994	3.338	0.980	−0.003	0.994	0.478	0.578
CMMTB11	0.974	0.101	0.974	−0.101	0.988	3.081	0.982	−0.002	0.991	0.500	0.487
CMMTB21	0.967	0.09	0.967	−0.09	0.994	2.757	0.988	−0.002	0.993	0.491	0.473
CMMTB31	0.969	0.083	0.969	−0.083	0.995	2.560	0.988	−0.001	0.988	0.522	0.345
CMMTB41	0.976	0.087	0.976	−0.087	0.990	2.663	0.990	−0.001	0.979	0.631	0.034
CMMTB51	0.985	0.080	0.985	−0.080	0.982	2.42	0.990	−0.001	0.976	0.730	0.301

**Table 5:** Gastroprotective studies of various groups.

Control Groups	Ulcerative Index (mm^2^)	Protection %	Gastric Wall Mucus (µg/gm/tissue)	Myeloperoxidase (µmoles/min/mg tissue)
Native Control	0	0	92.50 ± 2.51	0.10 ± 0.02
Model Control	85.21 ± 3.12	-	54.50 ± 3.52	2.67 ± 0.09
Positive Control	10.26 ± 1.95	88.20	89.53 ± 2.83	0.60 ± 0.15
Low Dose	52.59 ± 2.84	38.23	68.56 ± 3.18	1.11 ± 0.08
High Dose	28.12 ± 2.15	66.94	75.51 ± 2.23	0.75 ± 0.12

**Table 6:** Composition of Silymarin-loaded Chitosan-MMT beads.

Ingredient	CMMTB10	CMMTB11	CMMTB21	CMMTB31	CMMTB41	CMMTB51
Silymarin (mg)	50	50	50	50	50	50
Chitosan (g)	1	1	2	3	4	5
MMT (g)	0	1	1	1	1	1
TPP (% w/v)	10	10	10	10	10	10
Glutaraldehyde(% w/v)	5	5	5	5	5	5
